# Phase III Trials of Standard Chemotherapy with or without Bevacizumab for Ovarian Cancer: A Meta-Analysis

**DOI:** 10.1371/journal.pone.0081858

**Published:** 2013-12-04

**Authors:** Mingyi Zhou, Ping Yu, Xiujuan Qu, Yunpeng Liu, Jingdong Zhang

**Affiliations:** 1 Department of Medical Oncology, The First Hospital of China Medical University, Shenyang, China; 2 Seven-Year Academic Program for Master, China Medical University, Shenyang, China; Kinghorn Cancer Centre, Garvan Institute of Medical Research, Australia

## Abstract

**Background:**

Platinum-based standard chemotherapy improves survival of ovarian cancer (OC), but the five-year survival rate remains below 50%. Antiangiogenic agents (7.5 or 15 mg/kg Bevacizumab, Bev) plus to standard chemotherapy improve progression-free survival (PFS) not overall survival (OS) in completed randomized controlled trials (RCTs). The efficacy and safety of two doses of Bev + standard chemotherapy remain controversial.

**Methods:**

MEDLINE, EMBASE, Cochrane Central Register of Controlled Trials, Cochrane databases and ClinicalTrials.gov were searched. The outcomes of eligible RCTs included PFS, OS and toxicities. Hazard ratio (HR) and relative risk (RR) were used for the meta-analysis and were expressed with 95% confidence intervals (CIs).

**Results:**

Bev + chemotherapy improved PFS (HR, 0.82; 95% CI, 0.75 to 0.89; *P* = .000) and OS (HR, 0.87; 95% CI, 0.77 to 0.99; *P* = .026) in newly diagnosed OC (2 trials, 2776 patients), and PFS (HR, 0.48; 95% CI, 0.41 to 0.57; *P* = .000) in recurrent OC (2 trials, 845 patients). Bev + chemotherapy increased non-CNS bleeding (RR, 3.63; 95% CI, 1.81 to 7.29; *P* = .000), hypertension grade ≥ 2 (RR, 4.90; 95% CI, 3.83 to 6.25; *P* = .000), arterial thromboembolism (RR, 2.29; 95% CI, 1.33 to 3.94; *P* = .003), gastrointestinal perforation (RR, 2.90; 95% CI, 1.44 to 5.82; *P* = .003), and proteinuria grade ≥ 3 (RR, 6.63; 95% CI 3.17 to 13.88; *P* = .000). No difference was observed between the two Bev doses in PFS (HR, 1.04; 95% CI, 0.88 to 1.24) or OS (HR, 1.15, 95% CI, 0.88 to 1.50), but 15 mg/kg Bev increased toxicities.

**Conclusion:**

Bev + standard chemotherapy delayed progression for newly diagnosed and recurrent OC, and improved survival for newly diagnosed OC. The 7.5 mg/kg dose appeared to be optimal for newly diagnosed OC patients with high risk for progression.

## Introduction

Each year, more than 200,000 women are diagnosed with advanced ovarian cancer (OC); over 100,000 die worldwide [1]. The five-year survival rate of OC remains below 50% [2]. Sequential therapies are employed to maximize length and quality of life. Despite good initial response to standard chemotherapy strategy (platinum and taxanes), most women suffer from disease progression and require further treatment. 

Tumor angiogenesis is pivotal in the development and progression of OC and is an ideal target for molecular treatment approaches [3,4]. Bevacizumab (Bev), a humanized monoclonal antibody that binds VEGF specifically, thus preventing activation of its receptors [5]. Bev has shown promise in many human solid tumors including colon [6], renal [7] and lung [8] carcinomas. Monk et al. first reported significant clinical benefit of Bev for patients with recurrent OC [5]. Based on this evidence, various studies investigated the efficacy and safety of Bev + standard chemotherapy in OC [9-17], which led to phase III randomized clinical trials (RCTs) that combined Bev with standard chemotherapy in postoperative patients with OC in the GOG-0218 [18], ICON7 [19], OCEANS [20], and AURELIA [21] studies. Although significantly longer progression-free survival (PFS) was shown in all studies, improvement in overall survival (OS) from Bev + standard chemotherapy was unconfirmed. These studies also varied in results for patients in different subgroups after stratification according to prognostic factors. Doses of Bev were 15 mg/kg in all studies, except for the ICON7 study in which the dose was 7.5 mg/kg, which raised the question of whether dose affects efficacy and safety. Thus, our meta-analysis evaluated efficacy and safety of the addition of Bev to standard chemotherapy, and different clinical benefits and toxicities between two doses.

## Methods

### Selection of Studies

The MEDLINE, EMBASE, Cochrane Central Register of Controlled Trials, Cochrane databases and ClinicalTrials.gov databases were independently reviewed from their dates of inception to July 2013 by Mingyi Zhou and Ping Yu, who searched on “ovarian neoplasms” and either “bevacizumab” or “Avastin.” Only human studies and RCTs published in English were eligible. Abstracts and information from conferences were also collected independently. Studies that met the following criteria were included: (1) prospective randomized phase III trials involving patients with OC after initial surgery; and (2) treatment with standard chemotherapy, with or without Bev. Quality assessment of papers was independently performed by us, who used the seven-point Jadad ranking system [22].

### Data collection

This meta-analysis evaluated PFS, OS and toxicities. The following information was extracted from each study: first author’s name, year of publication, trial phase, intervention, primary end point, and secondary end points. For PFS and OS, the hazard ratios (HRs) and confidence intervals (CIs; 95% in all cases cited here) were derived from each paper directly. PFS was calculated from randomization to disease progression or death; OS was calculated from randomization to death. Disease progression was defined according to the Response Evaluation Criteria in Solid Tumors (RECIST) criteria, global deterioration of health and increased CA-125 level without isolated progression. For toxicities, numbers and rates of events were extracted from papers. Toxicities were graded according to the Common Terminology Criteria for Adverse Events, version 3.0.

### Statistical analysis

Statistical analyses of pooled PFS, OS, and toxicities were performed with STATA 11.0 software. For PFS and OS, HRs and CIs derived from papers were pooled. For toxicities, relative risks (RRs) and CIs were calculated according to data derived from each paper. Statistical heterogeneity among trials was assessed with Cochrane’s *Q* statistic, and inconsistency was quantified with the *I* ^2^ statistic [100% × (Q – df)/Q] [23]. *P* > 0.05 was considered to indicate homogeneity. To pool the HRs and RRs, a fixed-effect model was used for homogeneity, and a random-effect model for heterogeneity. We also investigated whether clinical benefit of Bev + standard chemotherapy for newly diagnosed OC could be affected by different prognostic factors, such as cancer stage, residual lesion size, patient’s age, tumor grade, and performance status score. Regrettably, only stratified PFS were performed, as stratified HRs and CIs of OS were not published until now.

We also investigated whether the two Bev doses (7.5 mg/kg and 15 mg/kg) were significantly different in efficacy. We extracted crude data from papers and calculated HRs and CIs using a prespecified algorithm of the preferred calculations [24]. A χ^2^ test was used to compare adverse event rates between the two doses. Analyses were conducted using SPSS software, version 16.0. *P* < 0.05 was considered statistically significant.

## Results

### Patients

**Figure 1:**
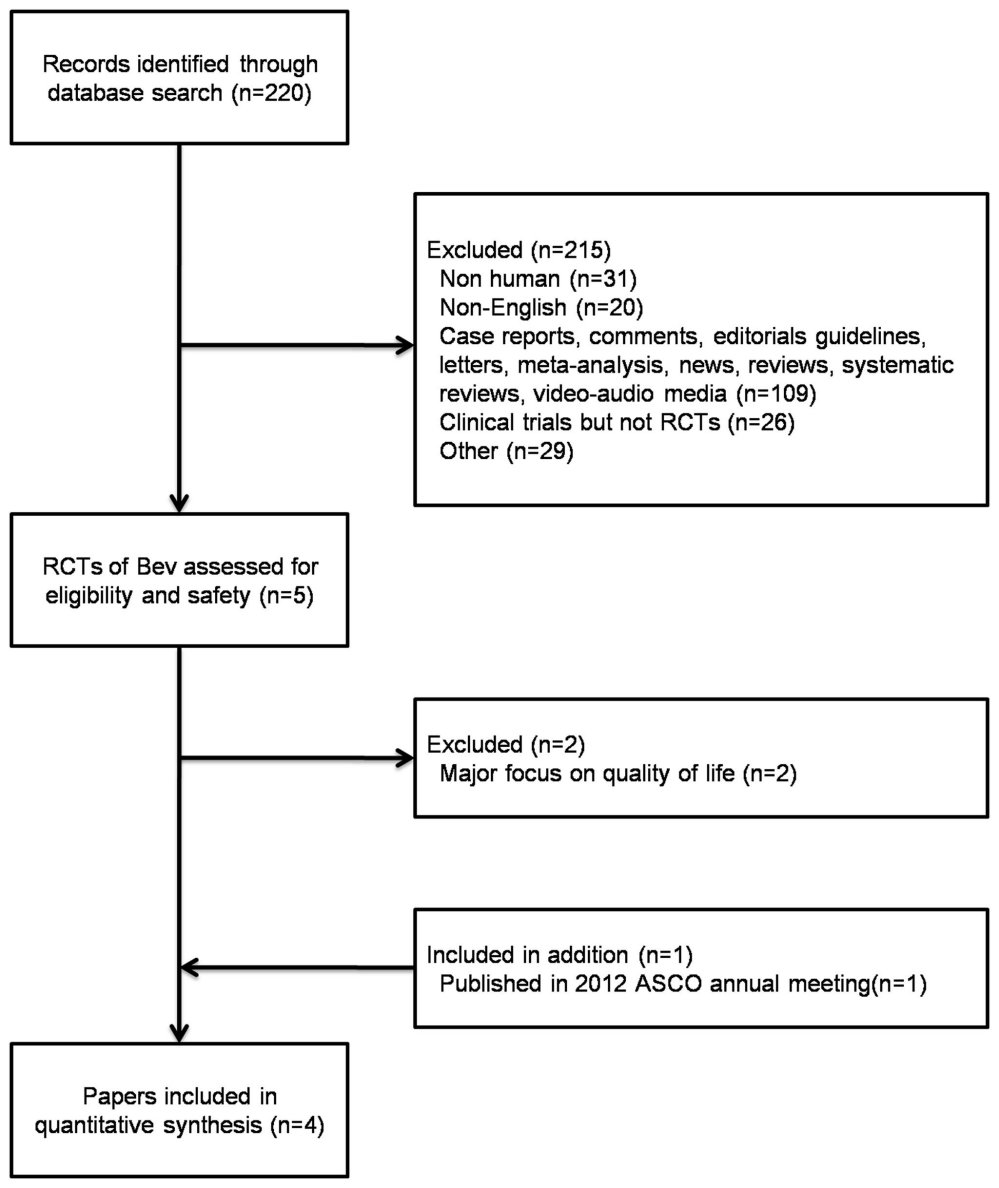
Selection process for randomized controlled trials included in the meta-analysis.

The 4 RCTs included here were randomized, multicenter, blinded, controlled phased III trials [18-21]. The GOG-2018 [18] and ICON7 [19] studies evaluated Bev + standard chemotherapy as adjuvant therapy for newly diagnosed OC after initial surgery. The OCEANS [20] and AURELIA [21] studies evaluated Bev + standard chemotherapy in platinum-sensitive and platinum-resistant recurrent OC. Figure 1 detailed the selection process.

A total of 3,621 patients were considered in the meta-analysis, of whom 1,808 received Bev + standard chemotherapy, and 1,813 received standard chemotherapy only. In the Bev + standard chemotherapy arm, although both Bev-initial therapy (Bev added in cycles 2–6) and Bev-throughout therapy (Bev added in cycles 2–22) were performed in the GOG-0218 study, only patients who received Bev-throughout therapy were involved in our meta-analysis, for two reasons: (a) results of the GOG-0218 study showed significant clinical benefit in the Bev-throughout therapy arm rather than the Bev-initial therapy arm [18]; and (b) this decision reduced heterogeneity, as only Bev-throughout therapy was applied in the ICON7 study. Jadad scores of the 4 RCTs were 6–7 which meant they were papers with high quality. Details were shown in Table 1.

**Table 1:** Characteristics of 4 RCTs.

	GOG-0218 2011 ^[18]^	ICON7 2011 ^[19]^	OCEANS 2012 ^[20]^	AURELIA 2012 ^[21]^
Primary end point	PFS	PFS	PFS	PFS
Secondary end point	OS, QoL	OS, response rate	OS, ORR, median duration of response	ORR, OS, QoL, Safety and tolerability
Population (treatment/control)	1248 (623/625)	1528 (764/764)	484 (242/242)	361 (179/182)
Stratification				
Stage/debulking	Stage III ≤1cm or Stage III >1cm or stage IV	Stage I-III ≤ 1cm or I-III > 1cm or IV and inoperable stage III	Surgery at relapse or no surgery at relapse	NR
Age	<60 yr or 60-69 yr or >70 yr	<60 yr or 60-69 yr or >70 yr	<65 yr or ≥65 yr	<65 yr or ≥65 yr
Tumor grade	Grade 1 or Grade 2 or Grade 3	Grade 1-2 or Grade 3	NR	NR
GOG/ECOG PS	GOG PS 0 or GOG PS 1/2	ECOG PS 0 or ECOG PS 1/2	ECOG PS 0 or ECOG PS 1	ECOG PS 0 or ECOG PS 1/2
Time to start chemotherapy/ Plat.-free interval	Time post operation to start chemotherapy	Time post operation to start chemotherapy (<4 or >4 weeks after surgery)	Plat.-free interval (6–12 or >12 months)	Plat.-free interval <6 months
Chemo/Bev duration in experimental arm(s)	Cycles 1-6: Carboplatin AUC 6, Paclitaxel 175 mg/m^2^, Bev 15 mg/kg (starting in cycle 2), q3w; Cycle 7-22: Bev 15mg/kg q3w	Cycles 1-6/18: Carboplatin AUC 6, Paclitaxel 175 mg/m^2^, Bev 7.5 mg/kg, q3w	Carboplatin AUC 4, Gemcitabine 1000 mg/m^2^, Bev 15 mg/kg, q21d	Paclitaxel 80 mg/m^2^ day 1, 8, 15, 22 q4w, Topotecan 4 mg/m^2^ days 1, 8, 15 q4w (or 1.25 mg/m^2^, days 1-5 q3w), PLD 40 mg/m^2^ day 1 q4w, Bev 15 mg/kg q3w
Chemo duration in control arm(s)	Cycles 1-6: Carboplatin AUC 6, Paclitaxel 175 mg/m^2^, Placebo (starting in cycle 2), q3w; Cycle 7-22: Placebo q3w	Cycles 1-6/18: Carboplatin AUC 5/6, Paclitaxel 175 mg/m^2^, q3w	Carboplatin AUC 4, Gemcitabine 1000 mg/m^2^, Placebo q3w	Paclitaxel 80 mg/m^2^ day 1, 8, 15, 22 q4w, Topotecan 4 mg/m^2^ days 1, 8, 15 q4w (or 1.25 mg/m^2^, days 1-5 q3w), PLD 40 mg/m^2^ day 1 q4w

Abbreviations: AUC= area under curve; Bev=Bevacizumab; BP=blood pressure; GCIG=gynaecological cancer intergroup; GOG=gynaecological oncology group; NR=not reported; ORR=overall response rate; OS=overall survival; PD=progressive disease; PFS=progression-free survival; plat.=platinum; PS=performance status; QoL=quality of life; RECIST= Response Evaluation Criteria in Solid Tumors.

### PFS

**Figure 2:**
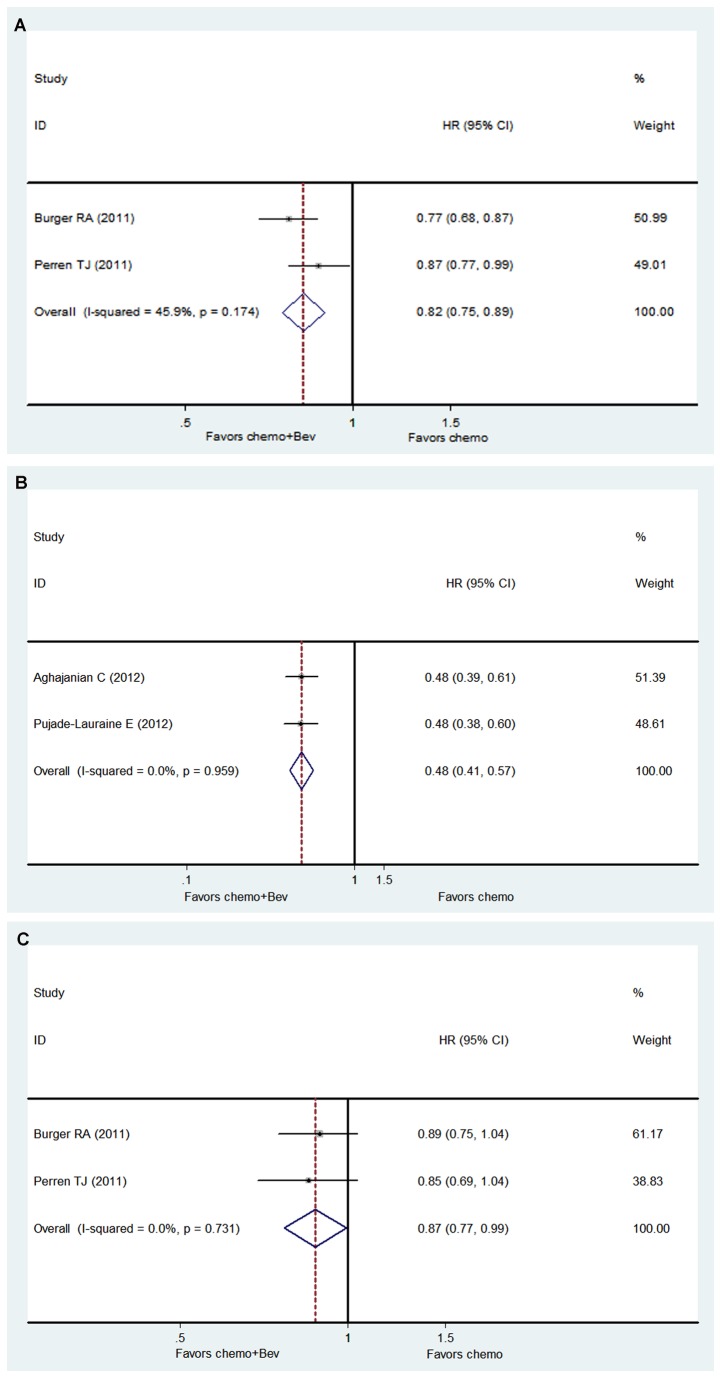
Hazard ratios (HRs) of progression-free survival and overall survival. (A) HRs of progression-free survival for GOG-0218 and ICON7; (B) HRs of progression-free survival for OCEANS and AURELIA; (C) HRs of overall survival for GOG-0218 and ICON7. Bev: bevacizumab; chemo: chemotherapy.

Improved PFS was seen with Bev + standard chemotherapy as adjuvant therapy for newly diagnosed OC after initial surgery (HR: 0.82, CI: 0.75 to 0.89, *P* = .000, fixed-effect model; GOG-0218 and ICON7 studies: 2776 patients, *I* ^2^ = 45.9%, *P* = .174; Figure 2A). Improved PFS was also observed in Bev + standard chemotherapy for platinum-sensitive and platinum-resistant recurrent OC (HR: 0.48, CI: 0.41 to 0.57, *P* = .000, fixed-effect model; OCEANS and AURELIA studies: 845 patients, *I* ^2^ = 0.0%, *P* = .959; Figure 2B).

### OS

Among patients with newly diagnosed OC after initial surgery, no significant difference was seen between the Bev + standard chemotherapy and control arms in GOG-0218 or ICON7 separately. However, improved OS was seen with Bev + standard chemotherapy after pooling HRs (HR: 0.87, CI: 0.77 to 0.99, *P* = .026, fixed-effect model; GOG-0218 and ICON7 studies, 2776 patients; *I* ^2^ = 0.0%, *P* = .731; Figure 2C). OCEANS found no significant difference in HR for OS between two groups (HR: 1.03, CI: 0.79 to 1.33; *P* > 0.05). As the OS endpoint was not achieved until now in AURELIA [21], we could not pool HRs of OS in recurrent OC.

### Subgroup analysis

**Figure 3:**
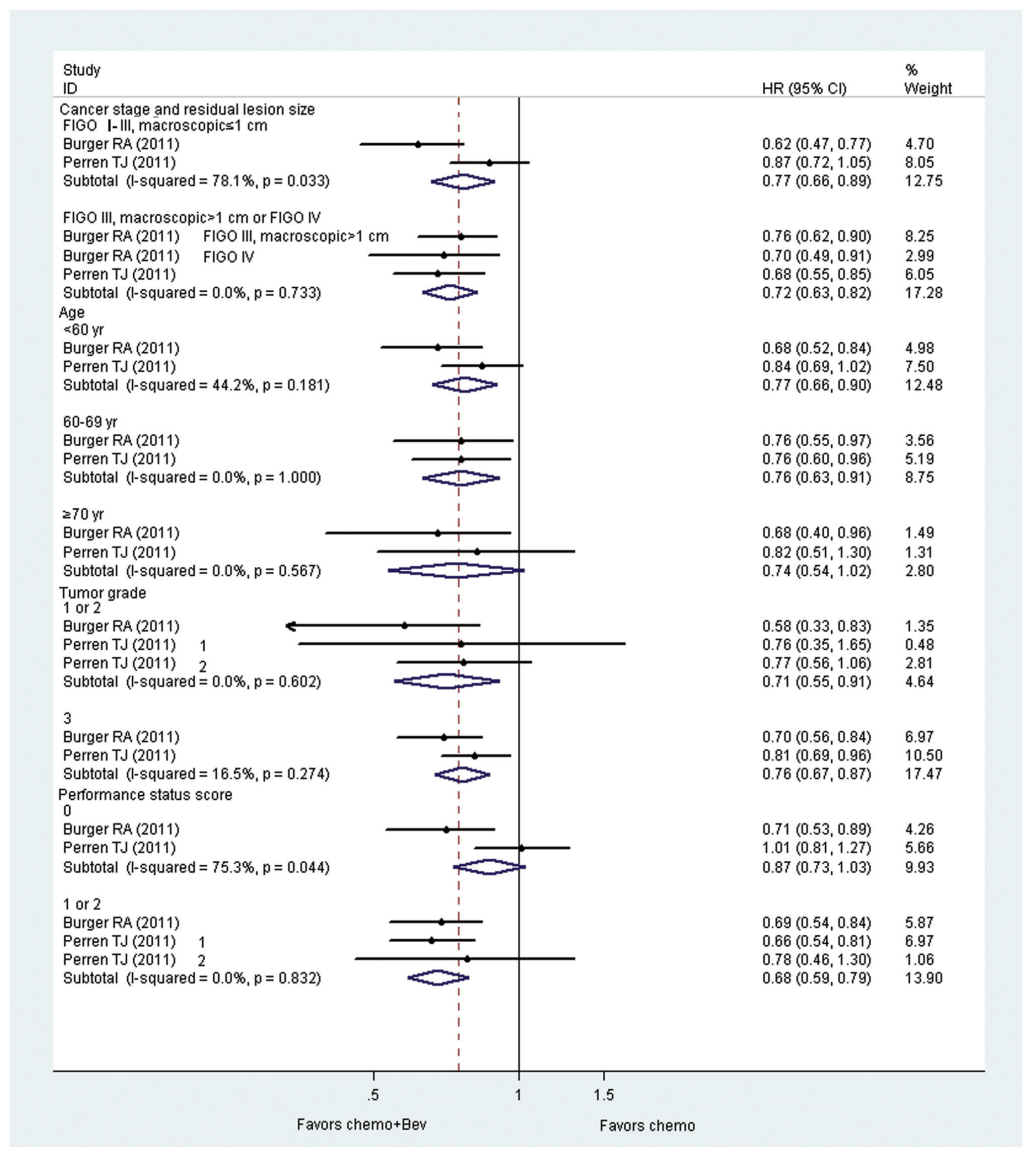
Progression-free survival by baseline risk factor. Bev: bevacizumab; chemo: chemotherapy; FIGO: International Federation of Gynecology and Obstetrics; HR: hazard ratio.

Estimation of the effect of Bev (vs. control) on PFS for newly diagnosed OC (GOG-0218 and ICON7) was stratified according to various prognostic factors (Figure 3). 

#### Cancer stage and residual lesion size

Both patients with high risk for progression (FIGO III, macroscopic >1 cm and IV) or with low risk for progression (FIGO I- II and III, macroscopic ≤ 1 cm) benefited from Bev + standard chemotherapy (high risk: HR: 0.72, CI: 0.63 to 0.82, *P* = .001; low risk: HR: 0.77, CI: 0.66 to 0.89, *P* = .001).

#### Age

Patients younger than 70 years benefited from Bev + standard chemotherapy (< 60 yr: HR: 0.77, CI: 0.66 to 0.90, P = .001; 60–69 yr: HR: 0.76; CI: 0.63 to 0.91, *P* = .003). However, no significant benefit was seen in patients aged 70 years or older (HR: 0.74, CI: 0.54 to 1.02; *P* = .067).

#### Tumor grade

Both patients at high tumor grade or at low tumor grade could benefit from Bev + standard chemotherapy (high grade: HR: 0.76; CI: 0.67 to 0.87, *P* = .000; low grade: HR: 0.71, CI: 0.55 to 0.91, *P* = .007).

#### Performance status score

Performance status score referred to patients’ general well-being, with healthier patients having lower scores. Thus, patients in poor condition benefited from Bev + standard chemotherapy (HR: 0.68, CI: 0.59 to 0.79; *P* = .000); whereas no significant benefit was observed in patients with well condition (HR: 0.87, CI: 0.73 to 1.03; *P* = .103). 

### Toxicities

**Figure 4:**
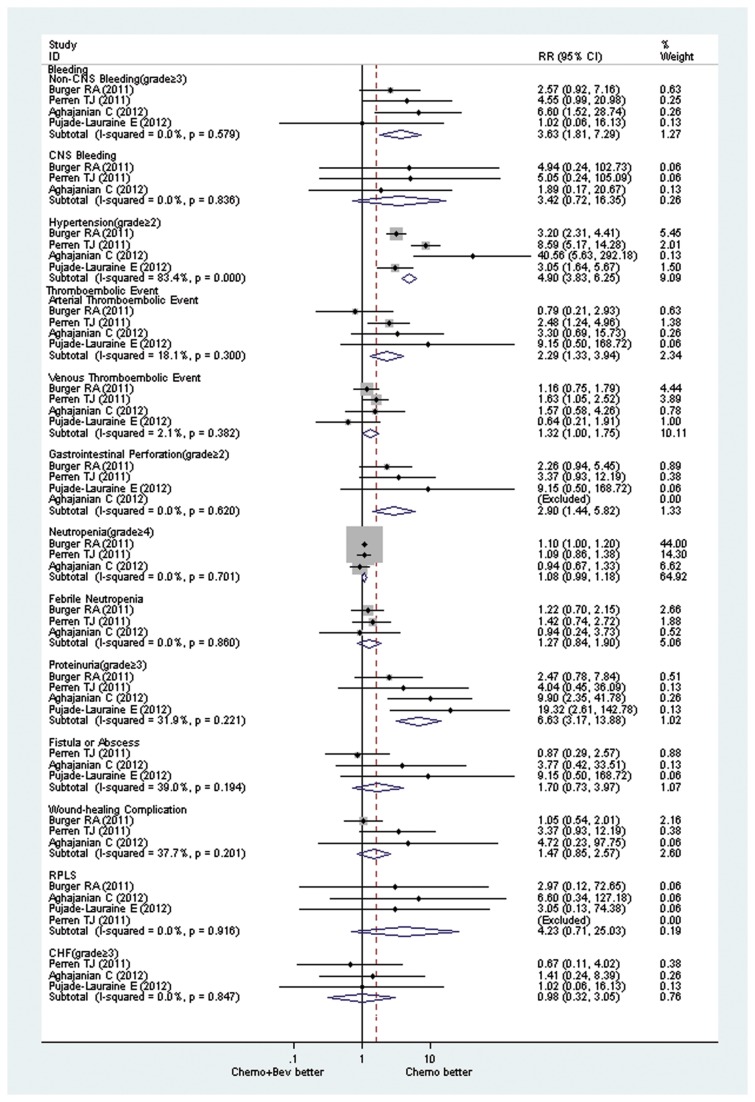
Relative risk of toxicities associated with bevacizumab + standard chemotherapy vs standard chemotherapy. Bev: bevacizumab; chemo: chemotherapy; CHF: congestive heart failure; CNS: central nervous system; RPLS: reversible posterior leucoencephalopathy syndrome; RR: relative risk.

Common toxicities related to Bev include hypertension, proteinuria, bleeding and arterial/venous thromboembolism (ATE/VTE). Frequency of occurrence and management of toxicities of Bev in a variety of other solid tumors has been described in detail [25]. Selected toxicities from the 4 RCTs were detailed in Table 2. Our meta-analysis found the addition of Bev led to greater risk for non-CNS bleeding (RR, 3.63; CI, 1.81 to 7.29; *P* = .000), hypertension of grade ≥ 2 (RR, 4.90; CI, 3.83 to 6.25; *P* = .000), ATE (RR, 2.29; CI, 1.33 to 3.94; *P* = .003), gastrointestinal perforation (GIP) (RR, 2.90; CI, 1.44 to 5.82; *P* = .003), and proteinuria of grade ≥3 (RR, 6.63; CI, 3.17 to 13.88; *P* = .000). The two arms showed no significant difference in the rates of other toxicities, including CNS bleeding, VTE, neutropenia of grade ≥ 4, febrile neutropenia, fistula or abscess, wound-healing complication, reversible posterior leucoencephalopathy syndrome, and congestive heart failure of grade ≥ 2 (Figure 4). 

**Table 2:** Selected adverse events for Bev in pivotal ovary cancer trials: GOG-0218, ICON7, OCEANS and AURELIA.

Clinical trial	interventions	Size of sample	non-CNS bleeding(grade≥3)		CNS bleeding		hypertension(grade≥2)		ATE		VTE		GIP(grade≥2)		Neutropenia (grade≥4)		febrile neutropenia		proteinuria(grade≥3)		fistula or abscess		wound-healing complication		RPLS		CHF(grade≥3)	
			No.	%	No.	%	No.	%	No.	%	No.	%	No.	%	No.	%	No.	%	No.	%	No.	%	No.	%	No.	%	No.	%
GOG-0218^[18]^	CP+Bev15->	608	13	2.1	2	0.33	139	22.9	4	0.7	41	6.7	16	2.6	385	63.3	26	4.3	10	1.6	NR	NR	18	3	1	0.2	NR	NR
	CP+placebo	601	5	0.8	0	0	43	7.2	5	0.8	35	5.8	7	1.2	347	57.7	21	3.5	4	0.7	NR	NR	17	2.8	0	0	NR	NR
ICON7^[19]^	CP +Bev7.5->	745	9	1	2	0.27	136	18	27	4	50	6	10	1	123	17	21	2.8	4	1	6	1	37	5	0	0	2	0.3
	CP	753	2	0.3	0	0	16	2.1	11	1.5	31	4	3	0.4	114	15	15	2	1	0.1	7	1	16	2.1	0	0	3	0.4
OCEANS^[20]^	CG+Bev15->	247	14	5.7	2	0.8	43	17.4	7	2.8	10	4.0	0	0	51	20.6	4	1.6	21	8.5	4	1.6	2	0.8	3	1.2	3	1.2
	CG+placebo	233	2	0.9	1	0.4	1	0.4	2	0.9	6	2.6	0	0	51	21.9	4	1.7	2	0.9	1	0.4	0	0	0	0	2	0.9
AURELIA^[21]^	CT+Bev	179	1	0.6	NR	NR	36	20.1	4	2.2	5	2.8	4	2.2	NR	NR	NR	NR	19	10.6	4	2.2	0	0	1	0.6	1	0.6
	CT	182	1	0.6	NR	NR	12	6.6	0	0	8	4.4	0	0	NR	NR	NR	NR	1	0.6	0	0	0	0	0	0	1	0.6
*I* ^*2*^ (%)			0.0		0.0		83.4		18.1		2.1		0.0		0.0		0.0		31.9		39.0		37.7		0.0		0.0	
RR			3.63		3.42		4.90		2.29		1.32		2.90		1.08		1.27		6.63		1.70		1.48		4.23		0.98	
(95%CI)			(1.81,7.29)		(0.72,16.35)		(3.84,6.25)		(1.33,3.75)		(0.995,1.82)		(1.45,5.82)		(0.99,1.18)		(0.84,1.90)		(3.17,13.88)		(0.73,3.97)		(0.85,2.57)		(0.72,25.03)		(0.32,3.05)	
*P*			0.000		0.123		0.000		0.003		0.054		0.003		0.086		0.256		0.000		0.217		0.169		0.112		0.976	

Abbreviations: ATE=arterial thromboembolism ; Bev=Bevacizumab 7.5 or 15 mg/kg; CG=carboplatin and gemcitabine; CNS=central nervous system; CHF=congestive heart failure; CP=carboplatin and paclitaxel; CT=chemotherapy; GIP=gastrointestinal perforation; NR=not recorded; RPLS=reversible posterior leucoencephalopathy syndrome; RR= relative risk; VTE=venous thromboembolism.

### Influence of Bev dose

Dose of Bev was applied in doses of 15 mg/kg in the GOG-0218 study and 7.5 mg/kg in the ICON7 study for patients with newly diagnosed OC. Results both in papers concluded, and our meta-analysis indicated, that patients with high risk for progression (FIGO III, macroscopic >1 cm and IV) were the major population who benefited from Bev + standard chemotherapy. Therefore, we attempted to investigate the efficacy and safety of Bev stratified by dose in patients with relative high risk for progression (FIGO III, macroscopic >1 cm and IV). Regrettably, the GOG-0218 study only provided the PFS and OS curves involved all patients with FIGO III-IV. So patients (FIGO III, macroscopic ≤ 1 cm) were not separated.

**Figure 5:**
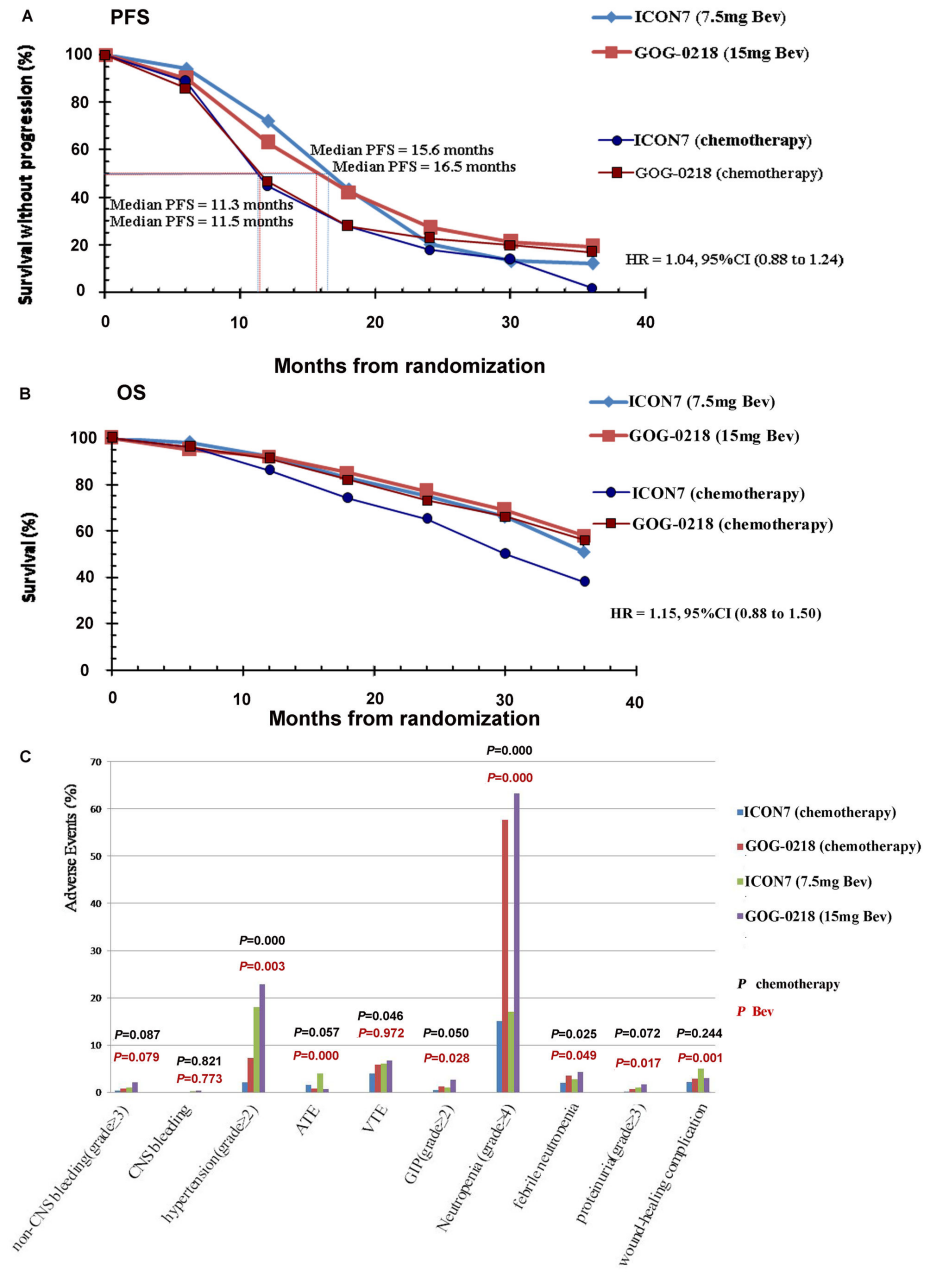
Comparison of the efficacy and safety of two dose of Bev. (A) progression-free survival curves; (B) overall survival curves; and (C) toxicity incidence between 7.5 mg/kg and 15 mg/kg Bev. Black *P*: toxicity incidence between ICON7 and GOG-0218 control arms; red *P*: toxicity incidence between 7.5 mg/kg and 15 mg/kg bevacizumab + standard chemotherapy arms. ATE: arterial thromboembolism; Bev: bevacizumab; CNS: central nervous system; HR: hazard ratio; GIP: gastrointestinal perforation; OS: overall survival; PFS: progression-free survival; VTE: venous thromboembolism.

PFS curves of patients stratified by Bev dose were shown in Figure 5A. For the control arms, median PFS were 11.3 and 11.5 months in ICON7 and GOG-0218 studies separately (HR, 1.14; CI, 0.96 to 1.34). For Bev + standard chemotherapy arms, median PFS was 16.5 months for patients with 7.5 mg/kg Bev and 15.6 months for patients with 15 mg/kg Bev (HR, 1.04; CI, 0.88 to 1.24).

The OS curves of patients stratified by Bev dose were shown in Figure 5B. Even though difference existed between the two control arms (HR, 1.60; CI, 1.24 to 2.06), no significant difference was shown between the two doses Bev + standard chemotherapy arms (HR, 1.15; CI, 0.88 to 1.50). 

Patients receiving 15 mg/kg Bev in the GOG-0218 study suffered more toxicities than with 7.5 mg/kg Bev in ICON7 study. No difference was shown between rates of GIP and proteinuria for patients in control arms of ICON7 and GOG-0218 studies (χ^2^ = 3.841, *P* = .050; χ^2^ = 3.233, *P* = .072); however, rates of GIP and proteinuria in patients receiving 15 mg/kg Bev were significantly higher than with 7.5 mg/kg Bev (χ^2^ = 4.833, *P* = .028; χ^2^ = 5.652, *P* = .017). Incidence of ATE and wound-healing complications were not consistent with other toxicities. Patients receiving 7.5 mg/kg Bev suffered more ATE and wound-healing complications than with 15 mg/kg Bev (Figure 5C).

## Discussion

For adjuvant therapy of newly diagnosed OC after initial surgery (ICON7 and GOG-0218 studies), Bev + standard chemotherapy reduced progression risk by 18%. For recurrent OC after platinum-based chemotherapy (OCEANS and AURELIA studies), Bev + standard chemotherapy reduced progression risk by 52%. The difference between reduction rates suggested that patients with recurrent OC achieved more benefit. For adjuvant therapy in newly diagnosed OC after initial surgery (ICON7 and GOG-0218 studies), Bev + standard chemotherapy reduced death risk by 13%. This result was inconsistent with the ones separately derived from ICON7 and GOG-0218 studies, in which significantly improved OS was shown in neither the primary analysis nor the updated analysis. Improved OS was also not observed in the meta-analyses published in 2011 [26] and March 2013 [27], because some updated data had not been taken into account. Several explanations for this variance occurred to us. Firstly, the updated data were not available when Gaitskell, et al. [26] performed their meta-analysis, but Ye and Chen [27] did not use the updated HRs of OS with unknown reason. Secondly, our meta-analysis amplified the sample size by pooling the data. Thirdly, this difference suggested that further prospective studies should be required to investigate if OS could be improved through Bev + standard chemotherapy. 

Additionally, our analyses included subgroups for newly diagnosed OC according to prognostic factors. Firstly, we found both patients with high risk and with low risk for progression benefited from addition of Bev, whereas in the ICON7 study, no benefit from addition of Bev was observed in patients with low risk for progression. This variance may be due to the different definitions of low risk for progression in the GOG-0218 and ICON7 studies. Not only patients defined as FIGO iii, macroscopic ≤1 cm but also patients defined as FIGO i-ii were placed in the ICON7 low risk subgroup. Secondly, we found patients who were younger than 70 years of age benefited from Bev + standard chemotherapy rather than patients who were 70 years old or older, and papers about other malignant tumors have reported that older patients suffered more toxicity and less benefit from Bev [28,29]. Thirdly, we found patients with both high and low tumor grades benefited from the addition of Bev, whereas ICON7 showed no benefit from the addition of Bev in patients with lower tumor grades. Fourthly, we found patients in poor condition benefited from addition of Bev rather than patients in good condition, which was consistent with results for non–small-cell lung cancer [30].

The addition of Bev was associated with higher incidence of toxicities (non-CNS bleeding, hypertension of grade ≥2, ATE, GIP, and proteinuria of grade ≥3) compared to standard chemotherapy. These toxicities were similar to those seen in other malignant tumors, such as metastatic colorectal cancer [6], non–small-cell lung cancer [30], and breast cancer [31-34].

The dose of Bev is another factor to be considered. For the GOG-0218 and ICON7 studies, in which patients had newly diagnosed OC, Bev doses were 15 mg/kg and 7.5 mg/kg respectively. Efficacy was very similar for the two doses. For PFS, difference existed neither between two control arms nor two doses Bev + standard chemotherapy arms. For OS, difference was shown between two control arms rather than two doses Bev + standard chemotherapy arms. Patients with low risk for progression (FIGO iii, macroscopic ≤ 1 cm) were also involved in the GOG-0218 study. So the benefit of OS of chemotherapy arms in the GOG-0218 study was more than the ICON7 study, and the two curves of the GOG-0218 study were not different. Curves involved just patients at high risk for progression from the GOG-0218 study were unavailable. Moreover, the OS for the patients at high risk for progression in the ICON7 study was less than all patients (patients at high risk for progression: HR, 0.64; CI, 0.48 to 0.85; all patients: HR, not yet reached) [19]. This suggested that the OS for the patients at high risk for progression would be less than all patients. No difference was shown in OS between the two doses Bev + standard chemotherapy. This indicated that, even though only patients at high risk for progression were involved, 15mg/kg Bev would not be demonstrated to prolong OS than 7.5 mg/kg Bev. For median PFS of patients who receive 7.5 mg/kg Bev in ICON7, the curve derived from the original paper (median PFS: 16.0 months) varied from the one shown in Figure 5A (16.5 months). However, for the control arm, the original paper reported 10.5 months and the curves in Figure 5A estimated at 11.3 months. Thus, trend and clinical significance was not influenced. Also, 15 mg/kg Bev carried more toxicity than did 7.5 mg/kg Bev except for ATE and wound-healing complications. Besides, the maintaining therapy of Bev was 9 months in the ICON7 study but 12 months in the GOG-0218 study. Considering toxicity and price, the 7.5 mg/kg dose is more cost-effective than 15 mg/kg Bev.

Finally, our meta-analysis was limited by the number of included papers, which were too few to power Begg’s and Egger’s tests for publication bias and sensitivity.

In conclusion, our meta-analysis suggested that Bev + standard chemotherapy delayed progression and improved survival for newly diagnosed ovarian cancer after initial surgery, and that addition of Bev delayed progression for recurrent ovarian cancer after platinum-based chemotherapy. We also found 7.5 mg/kg Bev to be an optimal dosage for newly diagnosed OC patients with high risk for progression.

## Supporting Information

Checklist S1 **PRISMA Checklist.**
(DOC)

Figure S1 **PRISMA Flow diagram.**
(DOC)
