# Supplementary material for: Phase III Trials of Standard Chemotherapy with or without Bevacizumab for Ovarian Cancer: A Meta-Analysis
Source: PLoS One. 2013 Dec 4;8(12):e81858. doi: 10.1371/journal.pone.0081858 (PMC3853655; doi:10.1371/journal.pone.0081858)
Supplement: Figure S1 — PRISMA Flow diagram. (DOC) [file pone.0081858.s002.doc]

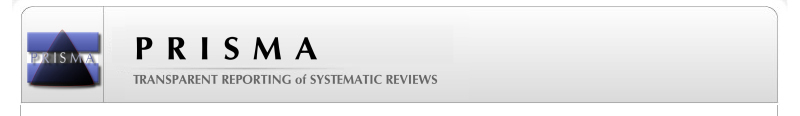
**PRISMA 2009 Flow Diagram**

**Screening**

**Included**

**Eligibility**

**Identification**

Records identified through database searching
(n = 220 )

Additional records identified through other sources
(n = 1 )

Records after duplicates removed
(n =221 )

Records screened
(n = 221 )

Records excluded
(n = 215 )

Full-text articles assessed for eligibility
(n = 6 )

Full-text articles excluded, with reasons
(n = 2 )

Studies included in qualitative synthesis
(n = 4 )

Studies included in quantitative synthesis (meta-analysis)
(n = 4 )
